# Supplementary material for: Dependence of aerosol-borne influenza A virus infectivity on relative humidity and aerosol composition
Source: Front Microbiol. 2024 Oct 16;15:1484992. doi: 10.3389/fmicb.2024.1484992 (PMC11521868; doi:10.3389/fmicb.2024.1484992)
Supplement: Supplementary file 1 [file Data_Sheet_1.pdf]

Supplementary Material

1 Supplementary Figures, Text and Tables

Supplementary Table S1. Composition of the media used in the present study.

Medium	Component	Concentration [g/L]
Phosphate-buffered saline (PBS)<br>Product number 18912 from ThermoFisher/Gibco	Sodium chloride	8.120
	Phosphate (as sodium phosphates)	0.950
	Potassium chloride	0.201
PBS + sucrose	Sucrose	64.0
	Sodium chloride	8.120
	Phosphate (as sodium phosphates)	0.950
	Potassium chloride	0.201

Supplementary Table S2. MIQE guidelines. Check-list of experimental details as requested by MIQE guidelines according to Bustin et al. (2009).

ITEM TO CHECK	PROVIDED Y/N	COMMENT
EXPERIMENTAL DESIGN		
Definition of experimental and control groups	Y	In materials and methods
Number within each group	Y	Specified in figures
SAMPLE		
Description	Y	In materials and methods
Microdissection or macrodissection	NA	
Processing procedure	Y	In materials and methods
If frozen - how and how quickly?	Y	In materials and methods
If fixed - with what, how quickly?	Y	In materials and methods

Sample storage conditions and duration (especially for FFPE samples)	Y	In materials and methods
NUCLEIC ACID EXTRACTION		
Procedure and/or instrumentation	Y	In materials and methods
Name of kit and details of any modifications	Y	In materials and methods
Details of DNase or RNAse treatment	Y	According to kit instructions
Contamination assessment (DNA or RNA)	Y	In materials and methods
Nucleic acid quantification	N	Not performed
Instrument and method	NA	
RNA integrity method/instrument	N	Not performed
RIN/RQI or Cq of 3' and 5' transcripts	NA	
Inhibition testing (Cq dilutions, spike or other)	Y	In materials and methods
REVERSE TRANSCRIPTION		
Complete reaction conditions	Y	In materials and methods
Amount of RNA and reaction volume	Y	In materials and methods
Priming oligonucleotide (if using GSP) and concentration	NA	
Reverse transcriptase and concentration	Y	According to kit instructions
Temperature and time	Y	In supporting information
qPCR TARGET INFORMATION		
Sequence accession number	N	Not provided
Amplicon length	Y	In materials and methods
In silico specificity screen (BLAST, etc)	NA	
Location of each primer by exon or intron (if applicable)	NA	
What splice variants are targeted?	NA	
qPCR OLIGONUCLEOTIDES		
Primer sequences	Y	In materials and methods

Probe sequences	Y	In materials and methods
Location and identity of any modifications	NA	
qPCR PROTOCOL		
Complete reaction conditions	Y	In materials and methods
Reaction volume and amount of cDNA/DNA	Y	In materials and methods
Primer, (probe), Mg++ and dNTP concentrations	Y	According to kit instructions
Polymerase identity and concentration	Y	According to kit instructions
Buffer/kit identity and manufacturer	Y	In materials and methods
Additives (SYBR Green I, DMSO, etc.)	Y	According to kit instructions
Complete thermocycling parameters	Y	In materials and methods
Manufacturer of qPCR instrument	Y	In materials and methods
qPCR VALIDATION		
Specificity (gel, sequence, melt, or digest)	N	Performed but not reported
For SYBR Green I, Cq of the NTC	N	Performed but not reported
Standard curves with slope and y-intercept	Y	In supporting information
PCR efficiency calculated from slope	Y	In materials and methods
r2 of standard curve	Y	In materials and methods
Linear dynamic range	Y	In materials and methods
Cq variation at lower limit	Y	In materials and methods
Evidence for limit of detection	Y	In materials and methods
DATA ANALYSIS		
qPCR analysis program (source, version)	Y	In materials and methods
Cq method determination	Y	In materials and methods
Outlier identification and disposition	NA	
Results of NTCs	N	Performed but not reported

Justification of number and choice of reference genes	NA	
Description of normalization method	NA	
Number and concordance of biological replicates	Y	Specified in figures
Number and stage (RT or qPCR) of technical replicates	Y	In materials and methods
Repeatability (intra-assay variation)	N	Not performed
Statistical methods for result significance	Y	In materials and methods
Software (source, version)	Y	In materials and methods

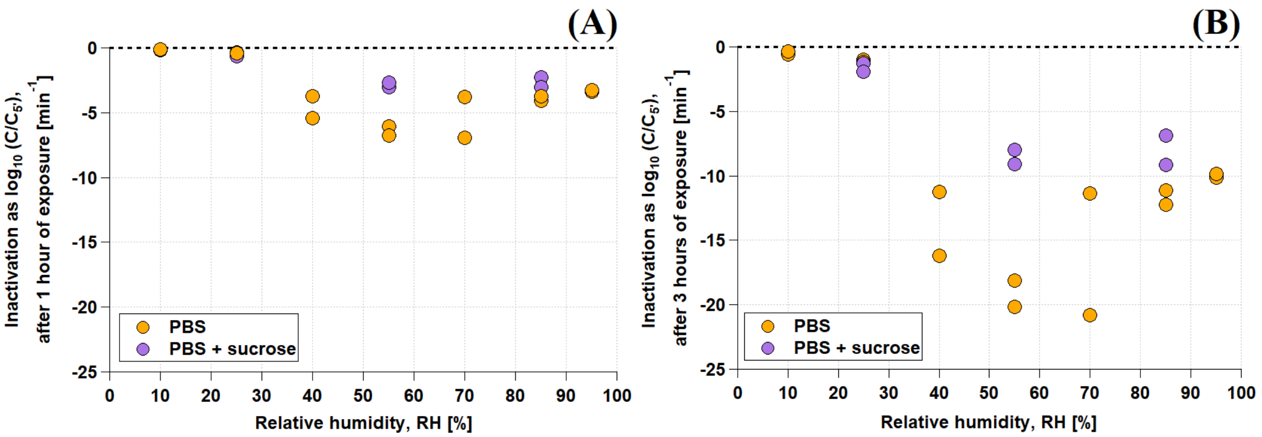

Supplementary Figure S1. IAV inactivation rate during the aerosol particle experiments performed in the LAPI BREATH (shown as t_{99} in Figure 3) after **(A)** 1 hour and **(B)** 3 hours of exposure.

Supplementary Text. Potential reasons for the discrepancy between aerosol particle and microliter droplet experiments

Figure 3 shows that IAV inactivation in 1-µL droplet and bulk experiments by Yang et al. (2012) and Schaub et al. (2023) using saline media is faster by up to an order of magnitude than inactivation in aerosol particles at medium and high RH. This cannot only be explained in terms of size effects. The equilibration between RH in the gas phase and a_w in the liquid phase, i.e., a_w = RH, is virtually instantaneous in submicron aerosol particles, whereas it typically takes less than 30 minutes in 1-µL droplets (Schaub et al., 2023). This could lead to differences of at most a factor of 2 (which has been correctly taken into account in the 1-µL droplet modeling). Furthermore, at RH > 50 %, there should be no efflorescence and potential complications resulting from the formation of complex dendritic morphologies can be avoided. Therefore, the observed large differences are surprising.

As mentioned in the main text of this paper, we can only speculate about the reasons for the discrepancies in t_{99}. Reasons could be the dependence of t_{99} on (i) the initial virus titer, (ii) the pH of

PBS, and (iii) exposure to air and strong surface tension forces that damage the viruses in/on the small aerosol particles but not in the large droplets. In the following, we qualitatively discuss some aspects of these potential reasons:

(i) **Dependence of t_{99} on the initial virus titer.** A virus can be affected by the presence of other viruses of the same strain, because they lead to the increase of organic molecules, which are known to have a protective effect (e.g., Kormuth et al., 2018), and possibly because viruses aggregate. While the initial titers in the bulk and droplet experiments were as high as 10^9 PFU/mL, we expect that aerosol particles in the LAPI BREATH will typically contain only one virus, if any, based on the work of Zuo et al. (2013) and Pan et al. (2019). To investigate whether this leads to different protection effects, we performed measurements in pure salt solutions with initial titers between less than 10^6 PFU/mL and more than 10^9 PFU/mL (see Figure S2). The corresponding t_{99} increases by a factor of 4 in this range of titer (as indicated by the slopes of the linear regression lines). We repeated these measurements for 0.14 M NaCl solution (in equilibrium with RH = 99.4 %) and found an even larger enhancement factor (not shown). Therefore, we hypothesize that a factor of 4 within the discrepancy in t_{99} might be due to differences in the initial titers between aerosol and large-volume measurements. This is illustrated by the yellow-shaded range in Figure S3.

(ii) **The dependence of t_{99} on the pH of PBS.** The pH value of a pure NaCl solution droplet stays constant during the drying process, namely close to pH 7. In contrast, in a PBS droplet, the partitioning between the ions (Na^+, Cl^-, H^+, $H_2PO_4^-$, HPO_4^{2-}) and their activity coefficients changes continuously during drying, which changes their pH. We measured the pH in PBS solutions at various concentrations and compared with our Pitzer ion interaction model (Luo et al., 2022) and found for 1 ´ PBS (a_w = 0.994) pH = 7.43 (measured) or pH = 7.39 (modeled) and for 20 ´ PBS (a_w = 0.898) pH = 6.58 (measured) or pH = 6.53 (modeled), corresponding to pH-induced reduction in t_{99} of about a factor of 3. Therefore, we hypothesize that with continuing drying of the PBS droplets, a growing fraction of the discrepancy in t_{99} between aerosol and large-volume measurements might be due to the concomitant pH reduction. This is illustrated by the red-shaded range in Figure S3.

(iii) **The dependence of t_{99} on tension forces.** Finally, we hypothesize that the viruses present in the aerosol particles (typically 100 nm in size), being exposed to air, develop triplet interface lines between the saline liquid, the gas phase and the organic phase of virus, and could therefore experience much stronger forces than in large droplets. This is due to higher surface tension effects on viruses, which are only partially covered by the saline solution, compared to complete immersion in the case of droplets. The surface tension on viruses contained within evaporating droplets may disrupt their envelope and inactivate them (Coleman et al., 2024). The same study concludes that osmotic pressure from dissolved salts still may dominate inactivation of viruses, not because of differences in their magnitude, but rather the exposure time to each force. Further efforts are needed to judge the importance of these effects.

From Figure S3 it is clear that the effects outlined in (i) to (iii) may explain the observed discrepancies to a large degree. However, without additional work, they remain speculative.

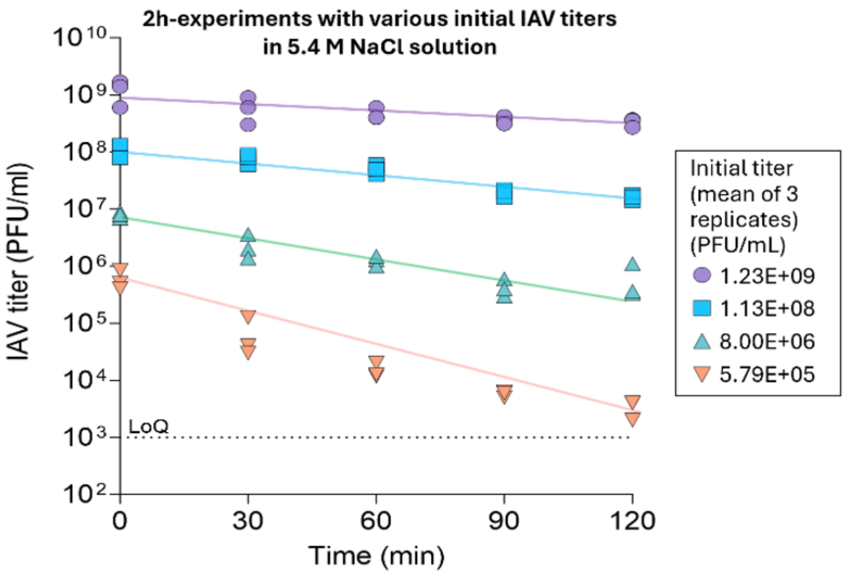

Supplementary Figure S2. Infectious IAV titer evolution in 5.4-M NaCl solution for different initial titers. Initial virus concentrations are indicated in the box on the right. The molarity of 5.4 M corresponds to a saturated solution, which is in equilibrium with RH = 74 % at room temperature.

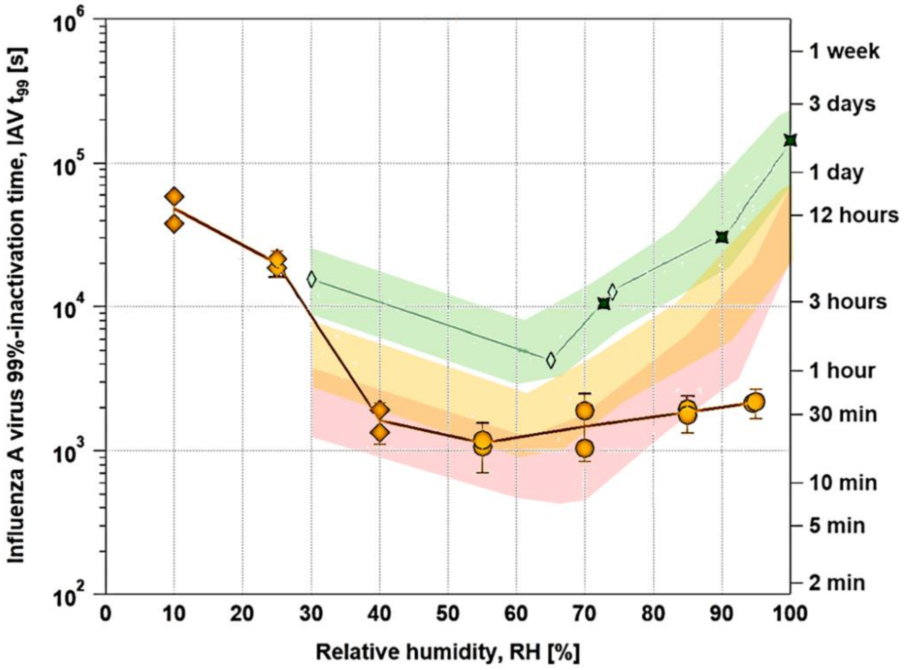

Supplementary Figure S3. Potential effects of high titer (yellow range) and pH dependence (red range), which might bring bulk and droplet measurements (green range) into agreement with aerosol measurements (orange circles). Processed RH-dependent IAV infectivity data in PBS from the LAPI BREATH (orange circles) in comparison with the pure NaCl solution measurements from Schaub et al. (2023) in bulk (green stars) and in 1-µL droplets (green diamonds). The high titer in the bulk

experiments may explain a factor of 4 of the discrepancy (from green range to yellow range) and the dependence of pH an additional RH-dependent fraction of the discrepancy (from yellow range to red range).
